# Mental Health Screening in Prison: Psychometric Evaluation of the PHQ-9 and GAD-7 Among Incarcerated Men in Mexico

**DOI:** 10.62641/aep.v54i2.2115

**Published:** 2026-04-15

**Authors:** Josué-Edgardo Hinojosa-López, Rebeca Robles-García, Sofía Rivera-Aragón, Damaris Chávez-Pedrote, Ana Fresán

**Affiliations:** ^1^Doctorate's student in Psychology, Master's and Doctorate Psychology Program of the National Autonomous University of Mexico (UNAM), Psychology Faculty, 04510 Mexico City, Mexico; ^2^Legal Medical Services and Social Reintegration Centers, Mexico City Ministry of Health, 06900 Mexico City, Mexico; ^3^Center for Research in Global Mental Health, Ramón de la Fuente Muñiz National Institute of Psychiatry, 14370 Mexico City, Mexico; ^4^Psychosocial Research Unit, Faculty of Psychology, National Autonomous University of Mexico (UNAM), 04510 Mexico City, Mexico; ^5^Laboratory of Clinical Epidemiology, Biomedical Research in Mental Health Directorate, Ramón de la Fuente Muñiz National Institute of Psychiatry, 14370 Mexico City, Mexico

**Keywords:** penitentiary centers, depression, anxiety, validity, reliability

## Abstract

**Background:**

Psychometric screening tools for persons in penitentiary centers (PPC) are key to assessing their mental health needs, given the shortage of both time and mental health personnel. Depression and anxiety are among the most prevalent mental health problems in PPC. Inadequate diagnosis can lead to increased symptom severity and even suicide. Therefore, validating instruments to assess depression and anxiety in PPC populations is essential to reduce the risk of misdiagnosis and its consequences.

**Methods:**

A cross-sectional study was conducted to evaluate the reliability and validity of the Patient Health Questionnaire-9 (PHQ-9) and the Generalized Anxiety Disorder-7 (GAD-7) in 272 men (>18 years old) deprived of liberty in a penitentiary center in Mexico. Both exploratory and confirmatory factor analyses were performed. Concurrent and discriminant validity were assessed using the depression and anxiety scales of the International Statistical Classification of Diseases and Related Health Problems, Eleventh Revision (ICD-11) Primary Health Care (PHC). Receiver Operating Characteristic curves were plotted to determine the best cut-off points in both instruments.

**Results:**

Both the PHQ-9 and GAD-7 showed items that were representative of their underlying constructs and supported the original one-factor structure, with satisfactory goodness-of-fit indices in both exploratory and confirmatory analyses. Both instruments exhibited good internal consistency in the sample of Mexican men incarcerated in penitentiary centers (α = 0.87 and α = 0.89, respectively). Furthermore, both scales showed strong correlations with the ICD-11 PHC depression and anxiety scales, along with adequate sensitivity, specificity, false positive, and false negative rates, resulting in a low misclassification rate.

**Conclusions:**

The PHQ-9 and GAD-7 can be used to briefly and reliably assess depression and anxiety among male PPC in Mexico City and in populations with similar institutional and sociodemographic conditions Given the specific characteristics of PPC populations across different countries, it remains necessary to continue testing these instruments in underrepresented populations to ensure the development of reliable and valid tools that help identify potentially confusing items and minimize diagnostic errors.

## Introduction

The number of persons in penitentiary centers (PPC) has increased by approximately 26% since the first decade of the 21st century [1]. When individuals are deprived of their liberty in penitentiary facilities, they nonetheless retain all fundamental rights and freedoms established in the Universal Declaration of Human Rights and the International Covenant on Economic, Social and Cultural Rights, including the right to the highest attainable standard of physical and mental health [2, 3].

The interaction between personal background, environmental conditions, and institutional factors contributes to poorer physical and mental health among PPC compared with the general population. Contributing factors include inadequate sanitary conditions [4], limited access to institutional activities, various forms of violence, and social isolation, among others [5, 6]. Given that mental health problems are also substantially more prevalent among PPC, penitentiary facilities have become some of the largest providers of mental health services [7, 8]. Therefore, ensuring adequate diagnosis and effective treatment is essential to improve mental health during incarceration, facilitate successful community reintegration, and reduce recidivism [9, 10, 11, 12].

Anxiety and depression are the most common mental health problems among PPC. Depression, a mental health condition characterized by persistent feelings of sadness, loss of interest in activities, and reduced energy or motivation, affects approximately 12.8% to 36.9% of this population and has been associated with sentence length, poor social support, and medical comorbidities [7, 13, 14, 15]. It is also linked to higher rates of self-harm, suicidal ideation, and suicide attempts, representing a major public health concern [16, 17, 18]. Anxiety, a mental health condition marked by excessive worry, nervousness, or fear that can interfere with daily activities, with an estimated prevalence of 36.1%, is more frequent among men aged 18–27 years and is associated with physical inactivity, chronic diseases, and sentences exceeding five years [19, 20]. Rates are up to three times higher in high-security prisons [21], and among men over 45 years, anxiety is related to greater functional impairment, multiple morbidities, and reduced physical health and quality of life [22].

Despite the high prevalence of mental disorders among PPC, penitentiary centers are not designed to provide mental health care. Consequently, mental health professionals are often scarce, and non-specialized personnel frequently conduct mental health assessments, creating significant gaps in diagnosis and adequate treatment [23, 24, 25]. Early detection of mental health problems in penitentiary settings is feasible and has been shown to significantly reduce psychopathology, improve long-term outcomes, and decrease care costs [26]. Mental health assessments in PPC are part of the minimum standards of care established for penitentiary institutions [27]. Nevertheless, institutional and environmental conditions within prisons can obscure or overlap symptoms of anxiety and depression. Therefore, valid and reliable clinical assessments are essential to enhance the accuracy of case detection, guide professional evaluation, and ensure access to appropriate and timely treatment. Moreover, validated instruments for assessing depression and anxiety in PPC can improve screening accuracy and facilitate adequate referrals to available mental health services [28, 29, 30].

The Patient Health Questionnaire-9 (PHQ-9) and the Generalized Anxiety Disorder-7 (GAD-7) are among the most widely used self-report instruments for the assessment of depressive and anxiety symptoms, respectively. Both scales have demonstrated solid psychometric properties, ease of administration, and sensitivity to change across a wide range of clinical and non-clinical populations [31, 32]. Their brevity and diagnostic alignment make them practical tools for large-scale screening, especially in resource-limited settings. However, despite their extensive validation in community and primary care samples, evidence regarding their psychometric performance in penitentiary populations remains scarce. Studies conducted in prison settings have primarily focused on prevalence estimation rather than formal validation, and some findings suggest that conventional cut-off points may overestimate symptom severity in incarcerated individuals [16, 17].

Although the PHQ-9 and GAD-7 are widely used tools for screening depression and anxiety, their clinimetric properties have not yet been evaluated in populations of incarcerated men in Mexico. Establishing the validity and reliability of these instruments in this specific context is essential, as incarcerated individuals face unique psychological stressors, environmental conditions, and social determinants that may influence the expression and measurement of mental health symptoms [33, 34, 35, 36]. Without proper validation, there is a risk of misdiagnosis or underdiagnosis, which can hinder access to adequate mental health care and the development of effective interventions within the prison system

Therefore, the objective of the present study was to evaluate the validity and reliability of the PHQ-9 and the GAD-7 scales in a sample of incarcerated men in Mexico. We hypothesized that both instruments would demonstrate adequate content validity, concurrent validity and discriminant validity, the latter assessed through comparison with the ICD-11 Primary Health Care (PHC) depression and anxiety screening tests, and a satisfactory reliability index.

## Methods

### Study Design and Participants

This cross-sectional study was conducted with a convenience sample of adult men (>18 years old) incarcerated in a penitentiary facility in Mexico City. Participation was voluntary and anonymous, and only individuals who provided informed consent were included.

The study was carried out in accordance with the ethical principles of the Declaration of Helsinki. All procedures and study materials were reviewed and approved by the Ethics Committee of the Master’s and Doctorate Program in Psychology in the National Autonomous University of Mexico (UNAM; approval EP/PMDPSIC/0095/2025), as well as by the administrative authorities of the correctional facility where recruitment was conducted. Participants who reported thoughts of death or suicidal ideation during the administration of the instruments were immediately referred for individualized evaluation by the correctional facility’s mental health team. This procedure ensured timely clinical assessment and appropriate follow-up in accordance with institutional mental health care protocols.

### Measures and Instruments

The PHQ-9 is a self-administered tool designed to assess the severity of depressive symptoms [32]. It comprises nine items, each aligned with the diagnostic criteria for major depressive disorder as defined in the Diagnostic and Statistical Manual of Mental Disorders, Fourth Edition (DSM-IV) and the Diagnostic and Statistical Manual of Mental Disorders, Fifth Edition (DSM-5), both widely used for the classification and diagnosis of mental disorders. Respondents rate the frequency of each symptom over the past two weeks on a four-point scale, from 0 (not at all) to 3 (nearly every day). Total scores range from 0 to 27, with higher scores indicating greater symptom severity. A score of 10 or higher is commonly used as a threshold for identifying moderate depression. Due to its brevity and ease of use, the PHQ-9 is widely implemented in both clinical and research settings as a reliable screening and monitoring tool. It has also been validated for use in the Mexican population, supporting its applicability across diverse cultural contexts [37, 38, 39].

The GAD-7 is a brief, self-administered screening tool used to assess symptoms of generalized anxiety disorder and to measure the severity of anxiety in clinical and research settings [40]. It includes seven items, each reflecting core symptoms of anxiety as outlined in the DSM-IV and DSM-5 diagnostic criteria. Respondents rate how often they have experienced each symptom over the past two weeks using a four-point Likert scale, ranging from 0 (not at all) to 3 (nearly every day). The total score ranges from 0 to 21, with higher scores indicating greater levels of anxiety. A score of 10 or higher is commonly used as a threshold for identifying moderate anxiety. The GAD-7 is widely valued for its simplicity, brevity, and strong psychometric properties, making it a useful tool for routine screening. It has demonstrated adequate reliability and validity for anxiety screening in the Mexican population [39, 41, 42].

The ICD-11 PHC screening test comprises five items each for the Anxiety Scale and the Depression Scale. A score of 3 or higher on either scale is considered indicative of clinically significant symptoms. In the Mexican population, this threshold has demonstrated a positive predictive value of 84% for identifying individuals with a current clinical diagnosis of depression, and 89.6% for those with a clinical diagnosis of anxiety [43]. Due to its brevity and strong predictive accuracy, the ICD-11 PHC screening test is a valuable tool for early identification of anxiety and depression in non-specialized settings.

Due to limited mental health resources in correctional facilities, individualized clinical interviews could not be conducted for all participants. The ICD-11 PHC guidelines provide standardized criteria for depression and anxiety and are designed for use in primary care and resource-limited settings. This makes the ICD-11 PHC an appropriate reference standard for validating screening instruments such as the PHQ-9 and GAD-7 in incarcerated populations.

### Procedure

From March 2025 to July 2025, participants were recruited during two mental health awareness events held at the penitentiary facility. Incarcerated men were invited to participate in the study, and those who expressed interest received a verbal explanation of the study procedures, the assessment instruments to be administered, and the estimated duration of participation (approximately 25 minutes). Individuals who provided verbal consent were scheduled for an individual assessment session in a private space specifically designated by the penitentiary for research purposes. All assessments were conducted by the facility’s mental health personnel, who were familiar with the project and trained in the administration of the instruments.

At the beginning of each session, participants were presented with a written informed consent form. They were also informed that all data would be treated as confidential and anonymous, and that the information collected would be used for research purposes. Any questions or concerns were addressed, and individuals who agreed to participate provided their written consent.

Demographic information (e.g., age, educational level, marital status) was collected, along with general legal background data, including the type of offense committed, legal status (pretrial or sentenced), and the length of incarceration at the time of the assessment. Participants then completed the PHQ-9, the GAD-7, and the ICD-11 PHC. Upon completion of the assessments, participants were thanked for their cooperation and provided with informational materials outlining available mental health resources within and outside the penitentiary facility, as part of a broader effort to support and promote their right to mental health care.

### Statistical Analysis

To summarize categorical variables, frequencies and percentages were calculated, while means and standard deviations (SD) were used for continuous variables. To assess the construct validity of the PHQ-9 and GAD-7, Exploratory Factor Analysis (EFA) was conducted on data from the first 136 participants using Principal Axis Factoring with maximum likelihood extraction method with a one-factor solution, based on the unidimensional design of both instruments. The Kaiser-Meyer-Olkin (KMO) measure of sampling adequacy and Bartlett’s test of sphericity were used to determine the appropriateness of the factor analysis. Items with factor loadings ≥0.40 were retained and assigned to the factor, in line with standard recommendations [44].

To confirm the exploratory structure identified in the EFA, Confirmatory Factor Analysis (CFA) was performed on the remaining 136 participants. Standardized factor loadings (i.e., standardized regression weights) of ≥0.40 were considered acceptable, indicating that items were adequately representative of the underlying latent construct [45]. CFA was conducted using maximum likelihood estimation, and model fit was evaluated using a range of commonly accepted fit indices [46]. A chi-square to degrees of freedom ratio (χ^2^/df) close to or below 3.0 was considered indicative of acceptable fit, the Root Mean Square Error of Approximation (RMSEA), with values <0.05 indicating good fit, 0.05–0.08 acceptable fit, and 0.08–0.10 marginal fit and the Comparative Fit Index (CFI) and Tucker-Lewis Index (TLI) with values ≥0.90 considered acceptable. Also, the Standardized Root Mean Square Residual (SRMR) was calculated with values <0.08 indicating good model fit. After the EFA and the CFA, internal consistency of the PHQ-9 and GAD-7 was determined.

A commonly recommended guideline for factor analysis is to have a sample size of 5 to 10 participants per item when conducting both exploratory and confirmatory factor analyses (EFA and CFA) to ensure stable and reliable factor solutions [47, 48]. It is essential to avoid using the same sample for both analyses, as each serves a distinct purpose and requires independent validation. EFA is employed to uncover the underlying factor structure of a set of variables without imposing a predefined model, whereas CFA evaluates how well a hypothesized factor structure fits the observed data. Using the same sample for both analyses may lead to overfitting and inflated estimates of model fit, compromising the generalizability of the findings. Therefore, two independent samples were formed by randomly assigning participants: one for EFA to explore the factor structure and another for CFA to confirm it, ensuring rigorous and reliable psychometric validation.

To assess concurrent validity, Pearson correlation coefficients were calculated between the PHQ-9 and the depression scale of the ICD-11 PHC, and between the GAD-7 and the anxiety scale of the ICD-11 PHC. Additionally, discriminant validity was evaluated using Receiver Operating Characteristic (ROC) curve analysis to identify optimal cut-off scores for both the PHQ-9 and GAD-7 in detecting moderate to severe levels of depression or anxiety. A score of 3 or higher on the ICD-11 PHC was used as the reference criterion. The cut-off score with the largest area under the curve (AUC) and the highest sensitivity and specificity were identified as the most accurate threshold for screening. PHQ-9 and GAD-7 quantify the severity of depressive and anxiety symptom dimensions that are empirically shown to underlie common mental-health presentations in primary care—the same dimensional constructs on which ICD-11 PHC diagnoses are based. Therefore, moderate or higher scores on these scales can be scientifically interpreted as reflecting clinically significant symptom burden that aligns with ICD-11 PHC disorder classifications.

All statistical analyses were conducted using SPSS version 21, IBM corp, Armonk, NY, USA and Stata/SE version 13.0 College Station, TX, USA: StataCorp LP for Windows. Statistical significance was set at *p* < 0.05.

## Results

### Demographic Description of the Study Population

A total of 272 incarcerated men participated in the study. The mean age of the sample was 36.7 (SD = 10.6, range 18–63) years. Almost half of the participants were married or partnered (47.4%, n = 129), followed by those who were single (41.2%, n = 112), 9.2% (n = 25) were divorced or separated and 2.2% (n = 6) were widowed. The average years of schooling were 8.2 (SD = 3.0, range 0–16) years. A little over 50% (51.5%, n = 140) were deprived of liberty for committing property-related offenses, followed by 39.0% (n = 106) for offenses against persons, 8.1% (n = 22) for drug-related offenses, and 1.5% (n = 4) for financial offenses. The length of incarceration at the time of the study was 23.4 (SD = 48.2, range 1–420) months. At the time of the evaluation, most participants were in pretrial detention (72.8%, n = 198) while the remainder (27.2%, n = 74) had already been sentenced.

### Exploratory Factor Analysis, Confirmatory Factor Analyses and Reliability of the PHQ-9 and the GAD-7 Questionnaires

In the EFA, the communalities of the PHQ-9 items ranged from adequate to excellent, with all values exceeding 0.40. These results indicate that each item shared a substantial portion of variance with the underlying factor, supporting their contribution to and adequate representation of the depression construct in the scale, and are consistent with the original one-factor solution of the questionnaire. The Kaiser-Meyer-Olkin measure of sampling adequacy was 0.80 with a significant Barlett’s sphericity index (*p* < 0.001) (see Table 1). Similarly, most of the GAD-7 items demonstrated adequate to excellent communalities, with most values exceeding 0.40. The only exception was the irritability item, which displayed a lower communality. Despite this, the item was retained in the CFAto assess its relevance and contribution to the anxiety construct, ensuring that potentially meaningful aspects of anxiety symptoms were not excluded from the evaluation. The KMO measure of sampling adequacy was 0.86, and Bartlett’s test of sphericity was significant (*p* < 0.001). The single-factor solution for the PHQ-9 explained 45.15% of the total variance, while the GAD-7 single-factor solution accounted for 57.71% of the variance. These proportions indicate that the extracted factors capture a substantial portion of the shared variance among the items in each scale, supporting their relevance in representing the underlying constructs of depression and anxiety, respectively. Although not all variability is accounted for, these levels of explained variance are considered meaningful in psychological research and provide a solid foundation for interpreting the PHQ and GAD as unidimensional measures of depressive and anxiety symptoms. The results of both factor analyses are presented in Table 1.

**Table 1.** **Exploratory factor analysis of the PHQ-9 and GAD-7 in incarcerated men**.

PHQ-9 items	Factor Loadings	GAD-7 items	Factor Loadings
Item 1		Item 1	
Interest or pleasure	0.623	Nervousness	0.737
Item 2		Item 2	
Depressed or hopeless	0.773	Control over worry	0.521
Item 3		Item 3	
Sleep changes	0.645	Worry	0.525
Item 4		Item 4	
Tired, lack of energy	0.705	Relaxation	0.744
Item 5		Item 5	
Appetite changes	0.414	Restlessness	0.426
Item 6		Item 6	
Worthlessness	0.875	Irritability	0.360
Item 7		Item 7	
Concentration changes	0.804	Fear	0.726
Item 8			
Psychomotor changes	0.70		
Item 9			
Self-harm, suicidal	0.408		
Variance explained	45.15%	Variance explained	57.71%

PHQ-9, Patient Health Questionnaire-9; GAD-7, Generalized Anxiety Disorder-7.

**Fig. 1.**
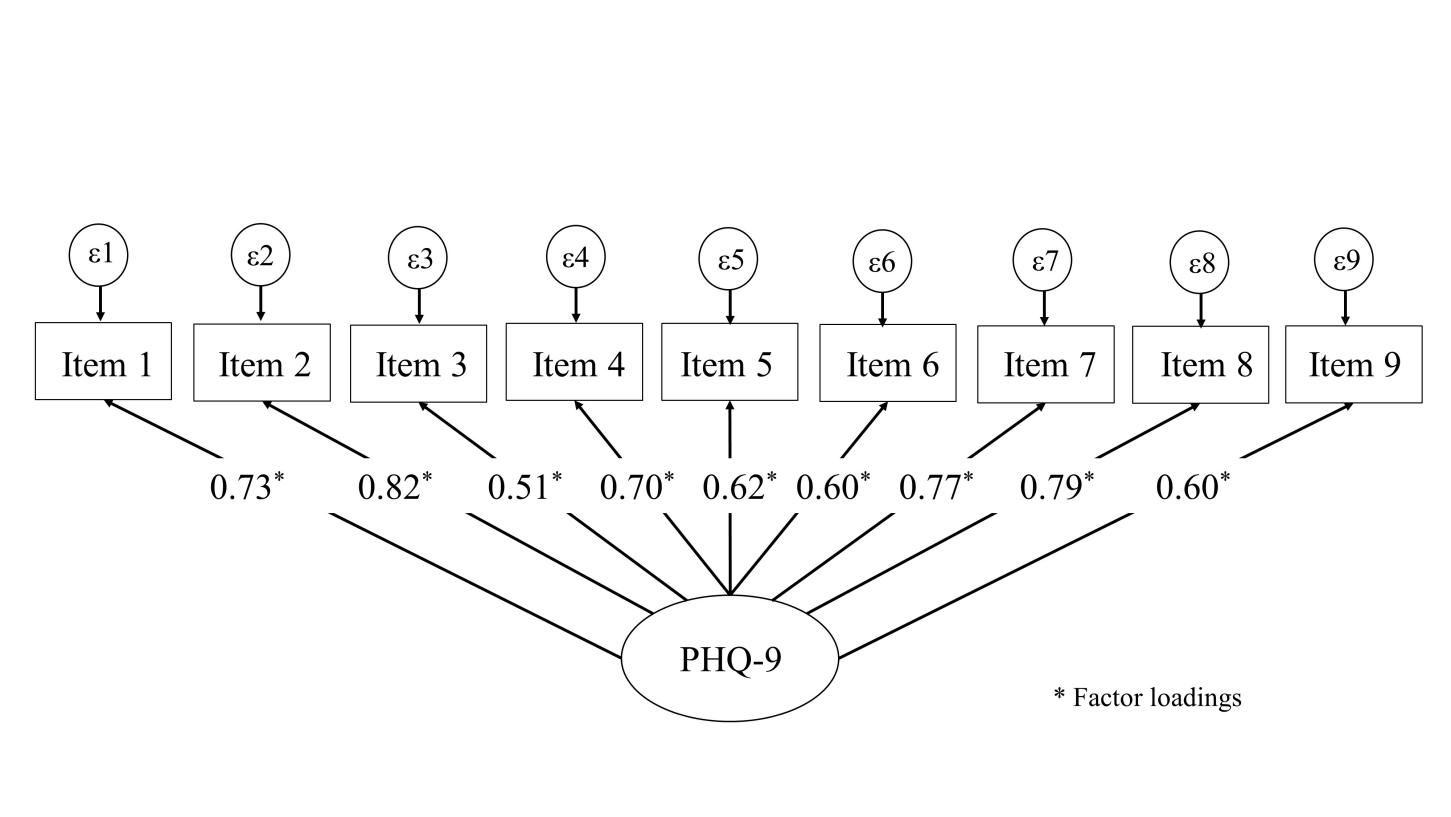
**Confirmatory factor analysis of the PHQ-9 in incarcerated men**. PHQ-9, Patient Health Questionnaire-9.

**Fig. 2.**
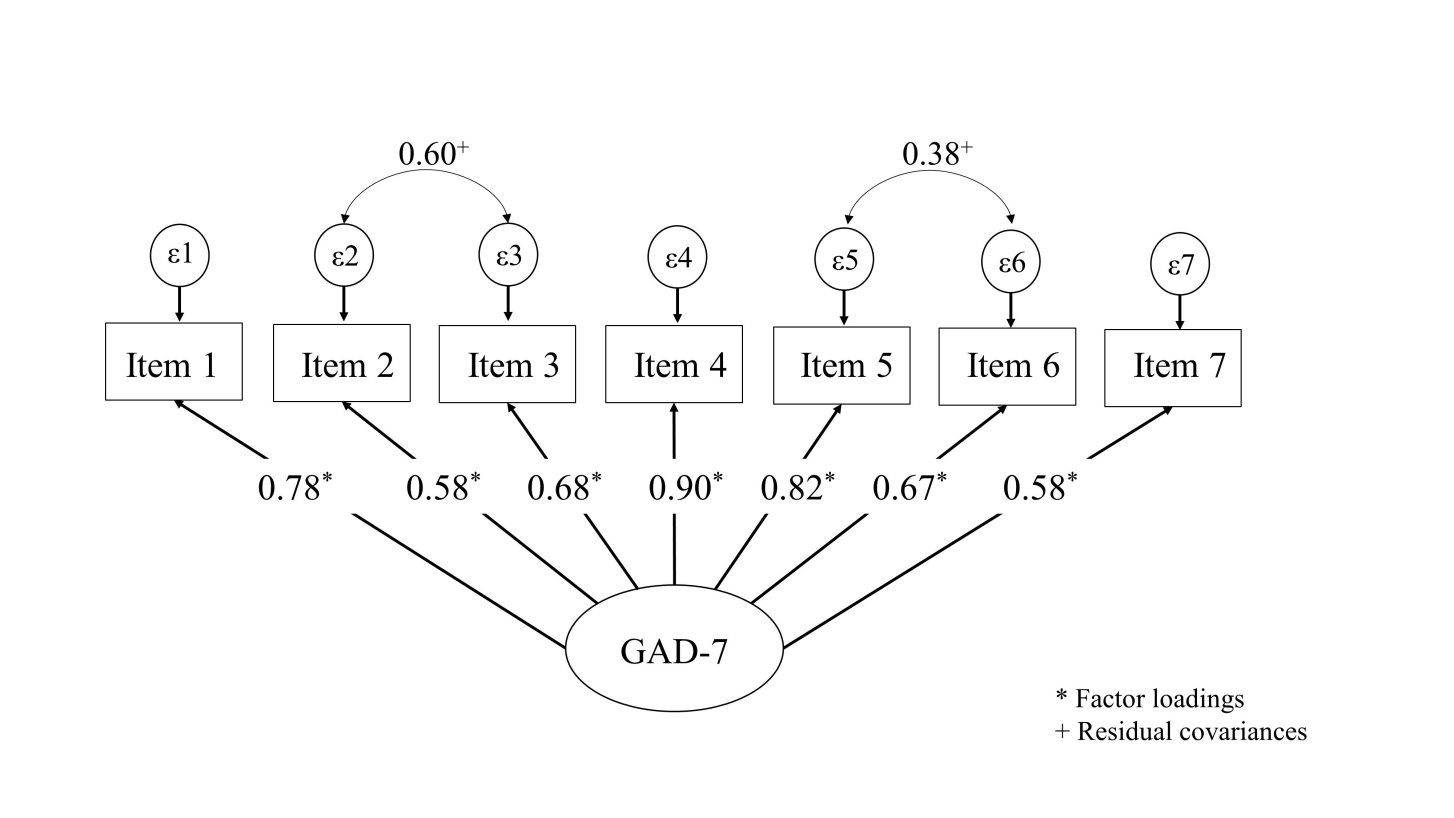
**Confirmatory factor analysis of the GAD-7 in incarcerated men**. GAD-7, Generalized Anxiety Disorder-7.

The CFA with the remaining 136 subjects, supported the unidimensional structure of both the PHQ-9 (see Fig. 1) and GAD-7 (see Fig. 2) questionnaires. All items demonstrated high standardized factor loadings, indicating strong associations with their respective latent constructs.

The PHQ-9 model yielded an RMSEA value of 0.09, suggesting a marginal fit, and an SRMR of 0.04, indicating a good fit. The chi-square to degrees of freedom ratio (χ^2^/df) was 2.1, while the CFI = 0.94 and TLI = 0.94 suggest an adequate model fit. No modification indices were suggested to improve this model.

The first CFA performed with the GAD-7 displayed inadequate goodness-of-fit-indices (χ^2^/df = 7.18; RMSEA = 0.21, CFI = 0.84, TLI = 0.76). Modification indices (MI) of the model suggested residual covariances between item 2 (control over worry) and item 3 (worry) and between item 5 (restlessness) and item 6 (irritability). By including these residual covariances the model improved significantly with adequate goodness-of-fit indices and a marginal fit displayed in the RMSEA value. The chi-square to degrees of freedom ratio (χ^2^/df) was 2.2, an RMSEA value of 0.09 and an SRMR of 0.03. Both the CFI = 0.97 and TLI = 0.95 were adequate.

Both questionnaires demonstrated adequate internal consistency, as assessed by Cronbach’s alpha. The PHQ-9 showed an alpha coefficient of 0.87, while the GAD-7 yielded an alpha of 0.89.

### Concurrent and Discriminant Validity of the PHQ-9 and the GAD-7 Questionnaires

Significant correlations were observed between the PHQ-9 and the ICD-11 PHC Depression Scale (r = 0.72, *p* < 0.001), as well as between the GAD-7 and the ICD-11 PHC Anxiety Scale (r = 0.62, *p* < 0.001), indicating good concurrent validity of both questionnaires.

**Fig. 3.**
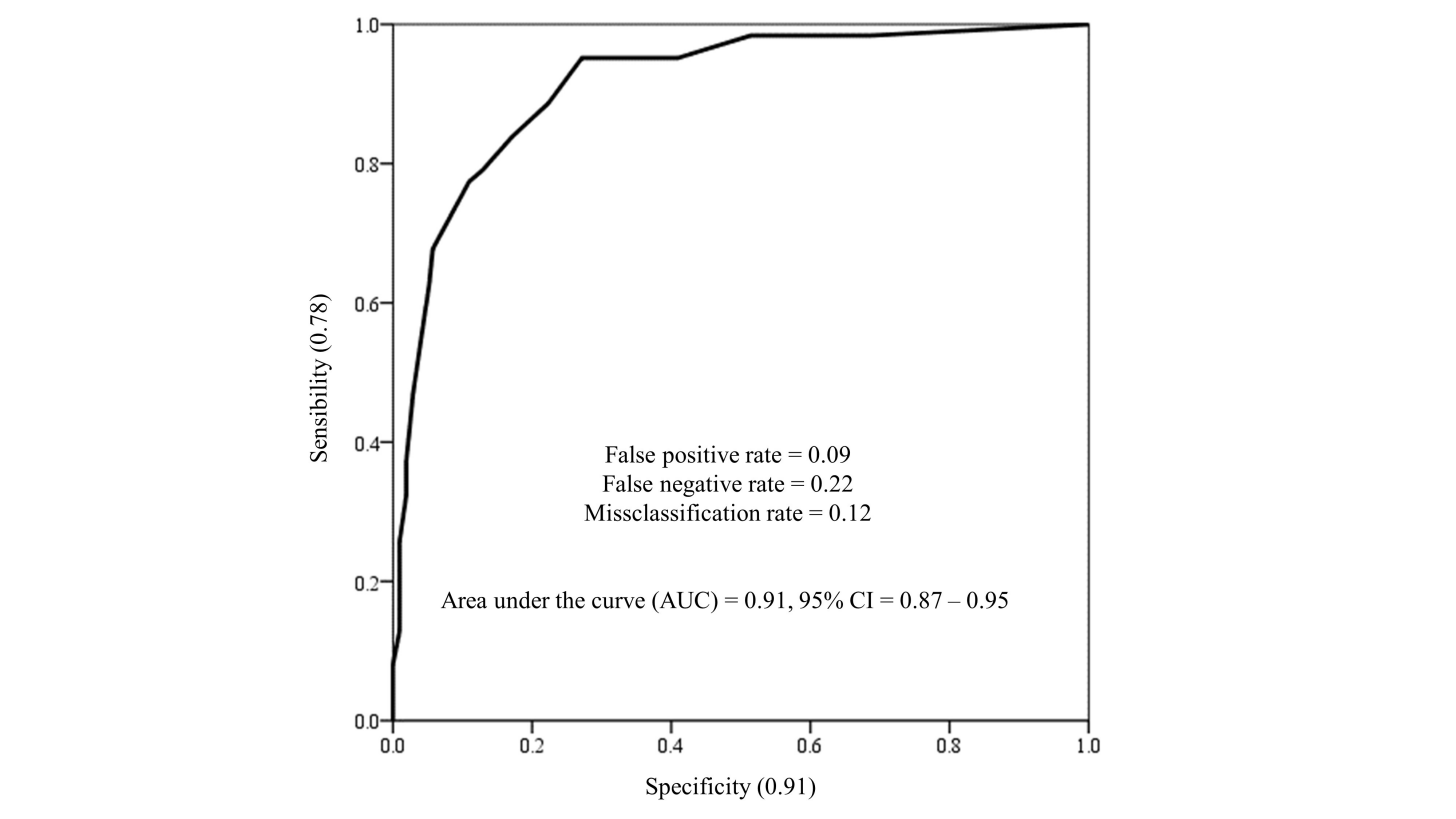
**ROC curve for the PHQ-9 using the ICD-11 PHC depression scale as reference**.

Regarding discriminant validity, the traditional PHQ-9 cut-off score of 10—commonly used to identify moderate depression—was the most effective in distinguishing participants with and without depression as defined by the ICD-11 PHC Depression Scale. This threshold demonstrated adequate sensitivity, specificity, false positive and false negative rates, a low misclassification rate, and an AUC exceeding 0.80 (see Fig. 3). According to this cutoff point, 19.9% (n = 54) of the participants reported moderate to severe depressive symptoms.

**Fig. 4.**
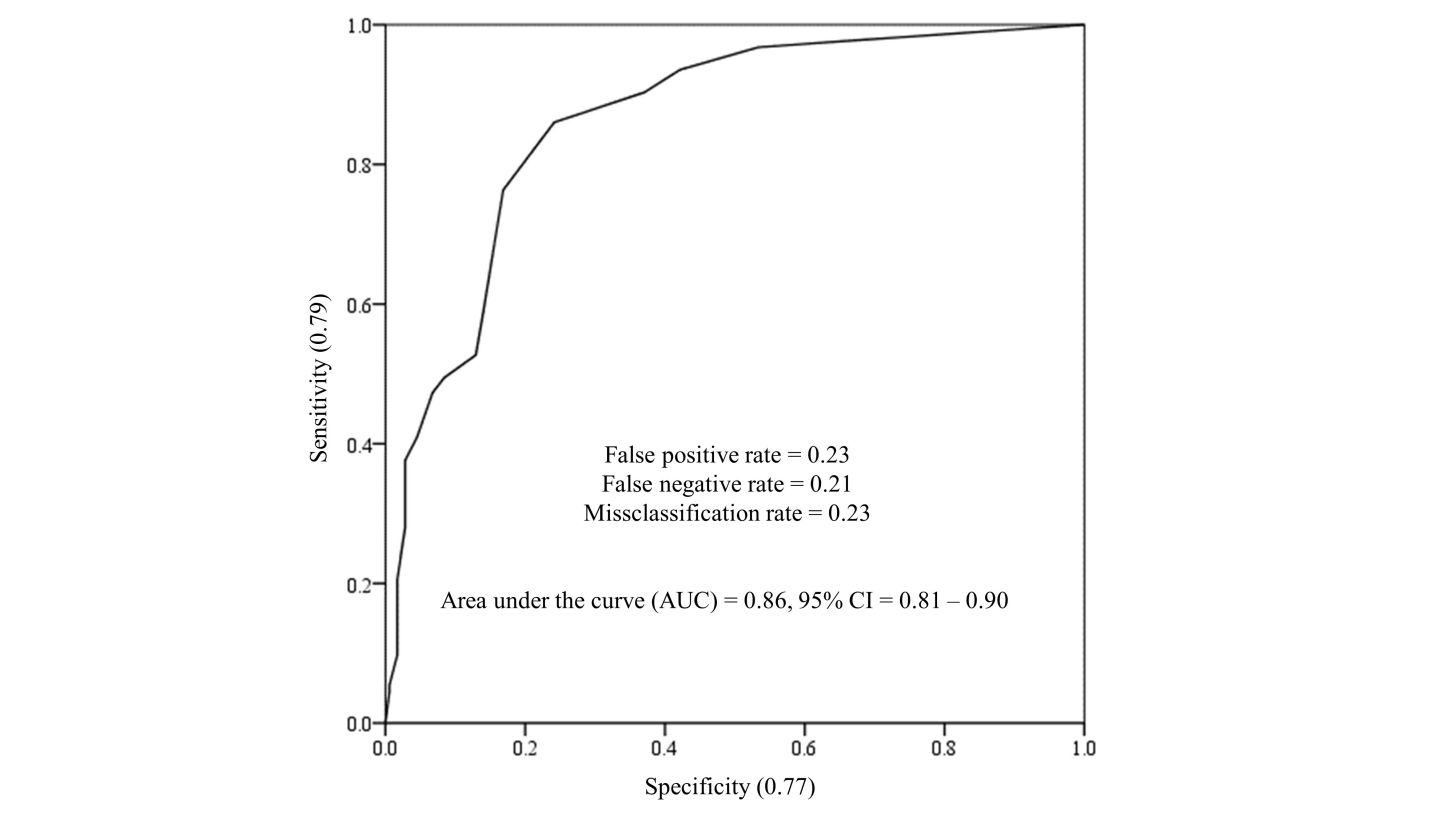
**ROC curve for the GAD-7 using the ICD-11 PHC anxiety scale as reference**.

Similarly, although the values were slightly lower than those observed for depression, the GAD-7 cut-off score of 10 showed the most appropriate diagnostic performance in identifying anxiety cases according to the ICD-11 PHC Anxiety Scale. It also yielded acceptable sensitivity, specificity, false positive and false negative rates, a low misclassification rate, and an AUC above 0.80 (see Fig. 4). According to this cutoff point, 20.6% (n = 56) of the participants reported moderate to severe anxiety.

## Discussion

The present study aimed to validate the PHQ-9 and GAD-7 questionnaires for use among PPC in Mexico. These instruments are widely used internationally for detecting depressive and anxiety symptoms in general and clinical populations [49, 50], and have also been applied successfully in correctional contexts [28]. However, to the authors’ knowledge, no prior validation had been conducted in Mexican PPC. Establishing their psychometric properties in this specific setting contributes to more accurate detection and diagnosis of depression and anxiety, reducing risks of under- or over-diagnosis, optimizing limited mental health resources, and upholding individuals’ right to health care [51, 52].

The EFA showed and exploratory one-factor structure for both scales, consistent with their original theoretical models. In the PHQ-9, all items loaded adequately on the latent factor, though item 9 (“thoughts of death or self-harm”) showed the lowest factor loading. This finding aligns with previous evidence suggesting that suicidal ideation may be underreported in penitentiary populations due to stigma, fear of consequences, and barriers to accessing specialized mental health care [17, 18, 25]. Likewise, the item assessing appetite changes exhibited a slightly lower factor loading, possibly reflecting contextual influences such as food quality and environmental conditions rather than depressive pathology [28].

The item assessing irritability in the GAD-7 (item 6) was retained for the CFA despite its low factor loading in the EFA. This decision was made because irritability is likely to be a particularly relevant symptom in the population under study—incarcerated men—where heightened stress, frustration, and interpersonal tension may manifest more prominently as irritability. Retaining this item allowed for a more in-depth examination of its behavior in a confirmatory context and provided the opportunity to assess whether it contributes meaningfully to the overall factor structure in this specific population. In the CFA of the GAD-7, the lowest loadings were observed for items 5 (“restlessness”) and 6 (“irritability”). These symptoms may overlap with depressive manifestations, which could explain their weaker association with the latent anxiety construct [28]. This weak correlation may also stem from the separation of ‘anxiety-related irritability’ and ‘environmentally triggered irritability’ in the prisoner population—the former is an emotional extension of anxiety, while the latter is an immediate response to incarceration-related oppression. Since GAD-7 Item 6 does not distinguish between these two mechanisms, its correlation with the latent anxiety construct is diluted. Despite these nuances, all items contributed meaningfully to the unidimensional factor structure, supporting the scales’ construct validity in this population.

CFA supported the unidimensionality of both instruments. The PHQ-9 demonstrated acceptable model fit indices without the need for modifications. In contrast, the initial GAD-7 model required the inclusion of correlated residuals between items assessing worry (items 2 and 3) and between restlessness and irritability (items 5 and 6) to achieve a marginal fit. This adjustment is theoretically justifiable, as worry and its perceived controllability are closely linked cognitive processes, while restlessness and irritability are often co-occurring physiological responses to anxiety. Additionally, differences in legal status (e.g., sentenced vs. awaiting sentencing) may influence the frequency and intensity of worry-related symptoms [28]. Therefore, the legal situation may represent a condition to consider when carrying out the evaluation.

In addition, the items assessing feelings of uneasiness (item 5) and irritability (item 6) showed improved factor loadings once the suggested modification indices were incorporated into the model. This adjustment enhanced the overall model fit and suggests that these symptoms are closely related constructs within this population. Importantly, irritability and restlessness may represent salient manifestations of anxiety in penitentiary contexts, where environmental stress, overcrowding, and limited autonomy can exacerbate emotional tension. Previous studies have reported that irritability is particularly frequent among PPC, especially in those with co-occurring personality disorders, with antisocial personality disorder being the most prevalent [9, 53]. Therefore, these symptoms should be carefully considered when interpreting anxiety measures in correctional populations, as they may reflect both underlying psychopathology and the influence of situational stressors inherent to incarceration.

It is important to note that modification indices were required to achieve the final result observed on the CFA of the GAD-7. Therefore, this analysis should be further examined in future studies to replicate these findings, which suggest a factor structure that may be less stable for use in incarcerated populations. Additional research would also provide more information on how best to interpret and apply this instrument in correctional settings.

Both scales showed excellent internal consistency, with Cronbach’s alpha coefficients above 0.85, comparable to previous studies in community and correctional samples [4, 28]. These findings indicate that the PHQ-9 and GAD-7 are reliable tools for assessing depression and anxiety symptoms among Mexican male PPC. Future research should extend this validation to female populations and other minority groups within correctional facilities to ensure broader applicability.

Concurrent validity was supported by the strong correlations between the PHQ-9 and the ICD-11 PHC Depression Scale, and between the GAD-7 and the ICD-11 PHC Anxiety Scale. These associations confirm that both instruments effectively measure their intended constructs. Moreover, discriminant validity analyses demonstrated that the conventional cut-off score of 10 provided optimal sensitivity and specificity for identifying clinically relevant cases of depression and anxiety, consistent with prior research [40, 54].

Several limitations of this study should be acknowledged. First, the sample included only male participants from one penitentiary center in Mexico, which represent a recruitment bias and restrict the generalizability of the findings to female inmates or to other correctional populations with different social and cultural characteristics. This could limit the generalizability of the findings to other prison settings or subgroups within the incarcerated population. Future studies with a larger number of participants from different correctional facilities, as well as the inclusion of female participants, are necessary to achieve greater generalizability of the results. Nevertheless, the present findings provide an initial foundation for the use of clinical instruments in correctional settings for the screening of anxiety and depression. Second, the cross-sectional design did not allow for the assessment of test–retest reliability or sensitivity to change, which are important aspects of psychometric validation in longitudinal monitoring or intervention contexts. Third, although the ICD-11 PHC scales were used as external criteria for concurrent validity, these instruments are screening tools rather than diagnostic interviews; therefore, future studies should include structured clinical assessments to confirm diagnostic accuracy. Fourth, environmental and situational stressors inherent to incarceration (e.g., overcrowding, noise, and security restrictions) as well as the assessment being performed during mental health awareness events at the penitentiary facility may have influenced symptom reporting, potentially inflating anxiety or depression scores. However, having validated clinical screening instruments for use in incarcerated populations is essential for the early identification of individuals at risk of mental health problems. Based on the results obtained from these tools, and through subsequent follow-up conducted by the correctional facility’s mental health team using specialized clinical interviews, it is possible to determine whether an individual is experiencing a depressive or anxiety disorder or whether the reported symptoms are secondary to the distress inherent to the incarceration context. Finally, self-report measures are subject to social desirability bias and the underreporting of sensitive symptoms such as suicidal ideation, particularly in prison settings where stigma and fear of repercussions remain significant barriers to disclosure [17, 18, 25].

However, our results suggest that the PHQ-9 and GAD-7 can be suitable for use in Mexican penitentiary contexts, where distinguishing genuine psychopathology from environmental distress is particularly challenging. The good diagnostic performance of these instruments may help reduce misclassification and improve access to appropriate treatment, ultimately mitigating adverse outcomes such as symptom worsening or suicidal behavior.

## Conclusions

Given the unique challenges of mental health assessment in correctional environments—such as limited resources, stigma, and procedural barriers—valid, reliable, and brief screening tools like the PHQ-9 and GAD-7 are essential. Their implementation could enhance early detection and referral processes, helping to close the existing mental health treatment gap in penitentiary centers [6, 50].

Implementing screening instruments such as the PHQ-9 and the GAD-7 in correctional facilities is highly useful for the timely detection and management of mental health problems. These tools allow for the efficient identification of individuals who may be experiencing clinically significant symptoms of depression or anxiety, facilitating early referral and follow-up by mental health professionals. Given the limited resources typically available in penitentiary settings, the use of brief, validated screening instruments contributes to optimizing clinical decision-making and prioritizing care for individuals at greater risk, thereby improving access to timely and appropriate mental health interventions.

The present validation provides robust evidence supporting the reliability and validity of the PHQ-9 and GAD-7 in Mexican male PPC. Developing and validating culturally and contextually adapted tools for diverse penitentiary populations remains a crucial step toward reducing diagnostic and treatment gaps. Integrating mental health assessment and care within state health systems—ensuring interdisciplinary and specialized services for individuals deprived of liberty—should be prioritized to protect their mental health and human rights.

## Availability of Data and Materials

The data presented in the manuscript is available on request from the corresponding author.
